# QuickStats

**Published:** 2013-03-08

**Authors:** Yelena Gorina

**Figure:**
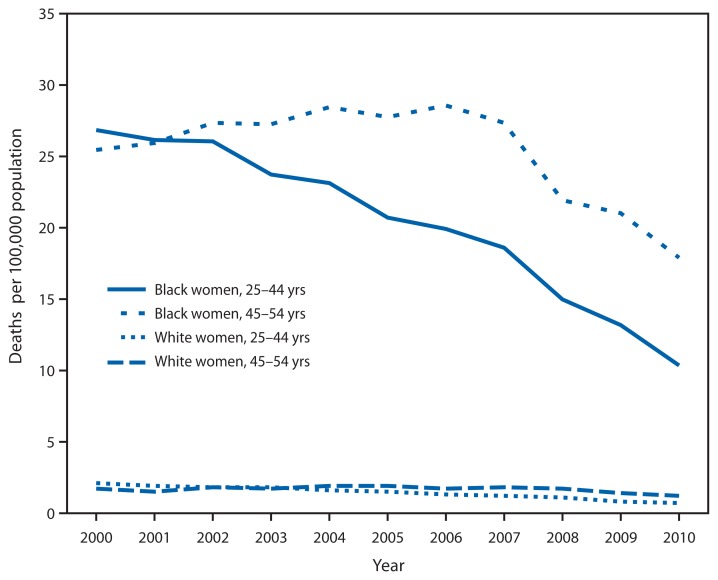
Human Immunodeficiency Virus (HIV) Disease Death Rates^*^ Among Women Aged 25–54 Years, by Race and Age Group — National Vital Statistics System, United States, 2000–2010 ^*^ Per 100,000 population. Deaths include those coded as B20–B24 in the *International Classification of Diseases, 10th Revision*.

From 2000 to 2010, HIV disease death rates decreased 61% for black women and 67% for white women aged 25–44 years. For women aged 45–54 years, the rates declined later in the decade. In that age group, rates decreased by 37% from 2006 to 2010 for black women and by 33% from 2007 to 2010 for white women. Throughout the 2000–2010 period, HIV disease death rates for black women were at least 12 times the rates for white women.

**Sources:** CDC. National Vital Statistics System. Available at http://www.cdc.gov/nchs/data_access/vitalstatsonline.htm.

